# Research advances of tissue-derived extracellular vesicles in cancers

**DOI:** 10.1007/s00432-023-05596-z

**Published:** 2024-04-10

**Authors:** Wei Li, Jingyao Zhu, Jiayuan Li, Yiyun Jiang, Jiuai Sun, Yan Xu, Hongzhi Pan, Yan Zhou, Jun Zhu

**Affiliations:** 1 Jiading District Central Hospital Affiliated to Shanghai University of Medicine and Health Sciences, Shanghai, 201800 People’s Republic of China; 2 Shanghai University of Medicine and Health Sciences, Shanghai, 201318 People’s Republic of China; 3 School of Materials Science and Engineering, Shanghai Jiao Tong University, Shanghai, 200240 People’s Republic of China; 4 Research Laboratory for Functional Nanomaterial, National Engineering Research Center for Nanotechnology, Shanghai, 200241 People’s Republic of China; 5 Shanghai University of Medicine and Health Sciences Affiliated Zhoupu Hospital, Shanghai, 200120 People’s Republic of China; 6 Department of Radiology, Renji Hospital, School of Medicine, Shanghai Jiao Tong University, Shanghai, 200127 People’s Republic of China

**Keywords:** Tissue-derived extracellular vesicles, Enzymatic digestion, Tissue culture, Density gradient centrifugation, Cancer diagnosis and treatment

## Abstract

**Background:**

Extracellular vesicles (EVs) can mediate cell-to-cell communication and affect various physiological and pathological processes in both parent and recipient cells. Currently, extensive research has focused on the EVs derived from cell cultures and various body fluids. However, insufficient attention has been paid to the EVs derived from tissues. Tissue EVs can reflect the microenvironment of the specific tissue and the cross-talk of communication among different cells, which can provide more accurate and comprehensive information for understanding the development and progression of diseases.

**Methods:**

We review the state-of-the-art technologies involved in the isolation and purification of tissue EVs. Then, the latest research progress of tissue EVs in the mechanism of tumor occurrence and development is presented. And finally, the application of tissue EVs in the clinical diagnosis and treatment of cancer is anticipated.

**Results:**

We evaluate the strengths and weaknesses of various tissue processing and EVs isolation methods, and subsequently analyze the significance of protein characterization in determining the purity of tissue EVs. Furthermore, we focus on outlining the importance of EVs derived from tumor and adipose tissues in tumorigenesis and development, as well as their potential applications in early tumor diagnosis, prognosis, and treatment.

**Conclusion:**

When isolating and characterizing tissue EVs, the most appropriate protocol needs to be specified based on the characteristics of different tissues. Tissue EVs are valuable in the diagnosis, prognosis, and treatment of tumors, and the potential risks associated with tissue EVs need to be considered as therapeutic agents.

## Background

Extracellular vesicles (EVs) is a collective term for vesicles with a membrane structure that are naturally released by nearly all the cells. Depending on their size and biological origin, EVs mainly include exosomes (EXOs), microvesicles (MVs), and apoptosis bodies (ABs). EXOs are intraluminal vesicles (ILVs) that arise from the inward budding of endosomal membrane during maturation of multivesicular endosomes (MVEs) and are secreted upon fusion of MVEs with the plasma membrane. MVs are derived from the outward budding and fission of the plasma membrane, liberating vesicles into the extracellular space. ABs, which are membrane-bound vesicles formed during the process of apoptosis and containing information and substances from dying cells, were discovered to be capable of delivering useful materials to healthy recipient cells (Raposo and Stoorvogel 2013; Colombo et al. 2014; Tkach and Théry 2016; van Niel et al. 2018).

EVs can be isolated from cell culture supernatants, body fluids, and tissues (Lozano-Ramos et al. 2015; Foers et al. 2018; Zhang et al. 2020a, b; Karimi et al. 2022) and play an integral role in multiple biological processes, including immune response, apoptosis, inflammatory response, and intercellular signal transduction (Kim et al. 2002; Peinado et al. 2012; Atay et al. 2014). They are, thus, involved in the pathophysiological events of most diseases, such as cancer, metabolic diseases, and neurodegenerative diseases (Yáñez-Mó et al. 2015; Kalluri and LeBleu 2020; van Niel et al. 2022). These make the work of isolation and characterizing of EVs attractive for the research in the fields recently.

Currently, isolated EVs can be divided into three types according to their sources, including cell culture-, body fluid-, and tissue-derived EVs (Ti-EVs). At present, most of the EVs studies focus on the cell culture- and body fluid-derived EVs. However, long-term culture may alter cell properties and subsequently affect the function of derived EVs (Allen et al. 2016). Cell lines may not be representative of the tumor from which they were derived either, due to missing the communication with the other co-existed cells in the tumor microenvironment (Domcke et al. 2013; Chen et al. 2015). Furthermore, EVs derived from cell culture are not able to reflect the dynamic progression of the disease over time, because the cell lines are obtained from one individual at a specific point during the development of the disease. In contrast, Ti-EVs are released by tissue cells under the strong influence of surrounding tissue cells and/or distant organs, and thus can provide comprehensive information on complex intercellular communication. And EVs obtained from tumor tissues resected from different patients better reflect the tumor heterogeneity from patient to patient than those from cell lines. For the identification of EVs biomarkers and true EVs function, Ti-EVs are superior to cell culture-derived EVs because they contain EVs secreted by most cells in the tissue and can reflect the pathophysiological characteristics and behavior of cells more accurately (Camino et al. 2020; Chen et al. 2020). Although EVs isolated from body fluids can reflect dynamic disease progression at a minimally invasive cost (Leung et al. 2021), they contain mixtures from various sources including serum proteins and huge amounts of lipoproteins (Simonsen 2017; Karimi et al. 2018; Wang and Turko 2018) or systemic EVs from the whole body (Jingushi et al. 2018). Meanwhile, the extent to which EVs are secreted from specific tissues into the circulation is still unknown at the moment (Huang and Xu 2021). While the Ti-EVs contained minimal contaminants due to the origin from only certain tissue compared to the EVs from body fluid (Crescitelli et al. 2020). This enables us to obtain highly pure organ-specific EVs for subsequent biomarker studies (Jingushi et al. 2018). In addition, Ti-EVs can be isolated from both cancer tissue and normal tissues nearby with a similar biological environment, and differences in Ti-EVs composition analysis from the same patient source are more beneficial for biomarker screening (Hoshino et al. 2020).

Due to the abovementioned characterization of the Ti-EVs, they play an important role in disease progression and have been found to have great potential in understanding the development and diagnosis of diseases in their early stages (Chen et al. 2022). Brain Ti-EVs are involved in central nervous system disease progression and thus can be used as biomarkers and therapeutic targets (Asai et al. 2015; Yelamanchili et al. 2015; Polanco et al. 2016; Wang et al. 2017; Ruan et al. 2021). Several studies have shown that proteins and RNAs in brain Ti-EVs are potential biomarkers for Alzheimer’s disease (AD) (Cheng et al. 2020; Huang et al. 2022). Adipose Ti-EVs play an critical role in the progression of obesity and insulin resistance, as well as some obesity-related metabolic diseases (Jayabalan et al. 2019; Camino et al. 2020). They can also promote wound re-epithelialization, granulation tissue formation, and hair follicle regeneration, thereby accelerating skin wound healing (Dai et al. 2017; Dong et al. 2020, 2022; Pan et al. 2023). More studies pay their attention on the role of Ti-EVs in tumors, and they have been shown to involve in various processes of tumorigenesis and progression. The tumor Ti-EVs have been used together with body fluid EVs for cancer early diagnosis, monitoring of treatment response, and analysis of unknown primary tumors (Hoshino et al. 2020; Tomiyama et al. 2021; Maruoka et al. 2022). Adipocytes are an important part of the tissue surrounding the tumor and adipose Ti-EVs were found to have widespread effects on tumors by providing metabolic substrates to support their progression and metastasis (Liu et al. 2023; Mathiesen et al. 2023). Exploring the role of adipose Ti-EVs in tumorigenesis and development can offer new strategies for tumor detection and treatment.

To fully make use of the Ti-EVs for understanding the development and diagnosis of diseases, isolation of Ti-EVs with a high purity is an important prerequisite. But this task remains highly challenging, mainly due to the complexity of Ti-EVs. There are few optimal methods readily available for the isolation and characterization of Ti-EVs that can meet experimental and clinical needs. This paper looks into the state-of-the-art technologies involved in the whole process of Ti-EVs isolation including tissue processing, Ti-EVs isolation and characterization, and shares our insights to facilitate the research in isolating and characterizing high-purity Ti-EVs. Then we provide an overview of the research progress of Ti-EVs for addressing the challenges raised by cancer management, including the role of Ti-EVs for tumorigenesis and progression, and the application of Ti-EVs for early diagnosis and treatment of tumors.

## Tissue processing

The interstitial space of solid tissues contains extracellular matrix (ECM), a complex network comprising diverse multi-domain macromolecules arranged in a cell- or tissue-specific configuration (Yue 2014). These ECM constituents intricately intertwine, engendering a robust composite structure that significantly influences the mechanical characteristics of tissues. The average mesh size of ECM is usually smaller than that of EVs (Meldolesi 2018; Lenzini et al. 2020), which makes EVs usually embedded in the ECM. The purpose of tissue processing is to release EVs confined in the ECM, or allow tissue cells to secrete more EVs by tissue culturing. It is also critical to minimize the impact on cellular integrity when working with tissues, so that the isolated vesicles are truly derived from the extracellular space and not intracellular vesicles or nanoparticles from the ruptured cells, thereby reducing the risk of contamination (Crescitelli et al. 2021). Note that even small numbers of dead cells are able to produce more vesicles than EVs (Théry et al. 2018).

**Fig. 1:**
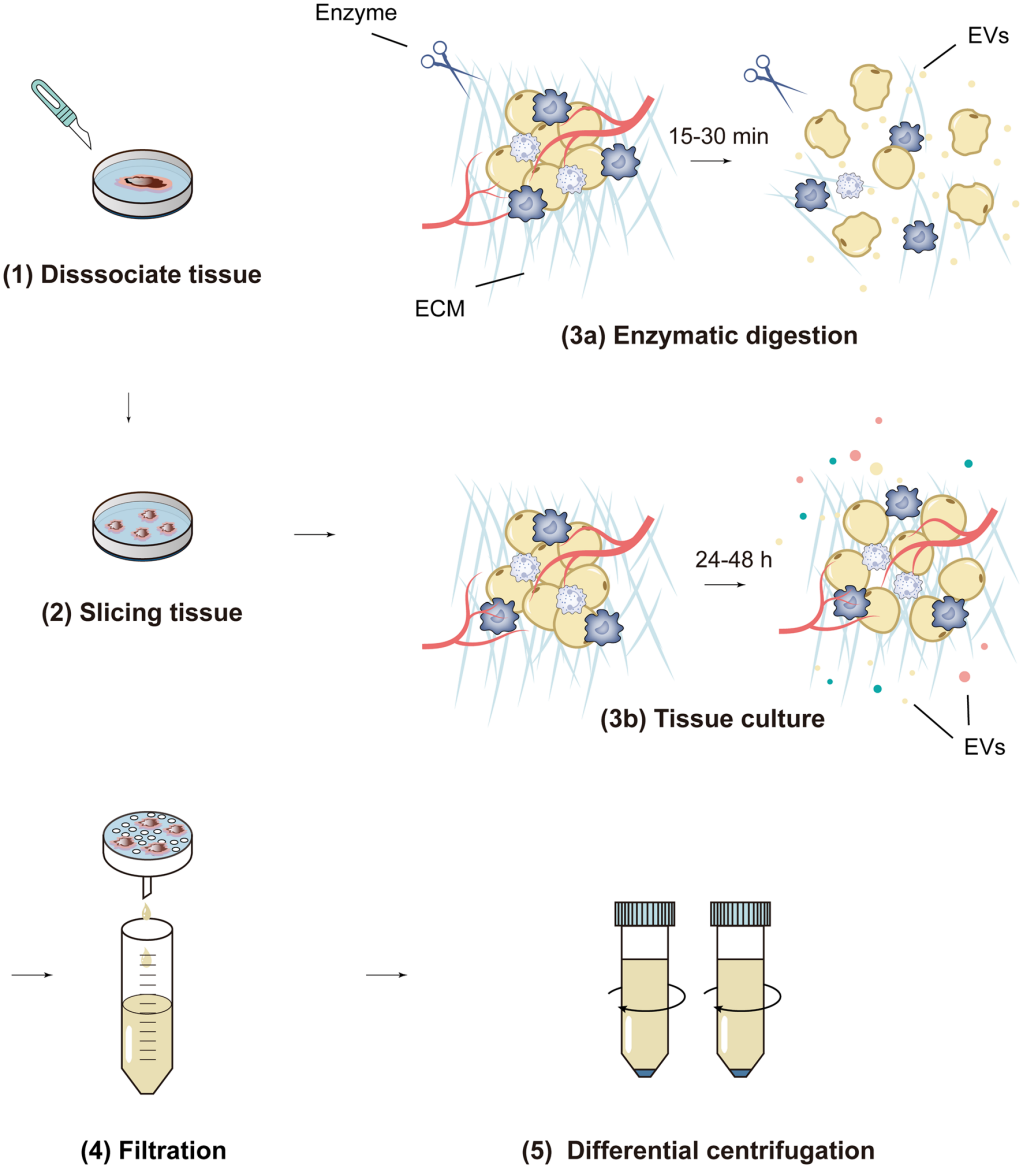
The tissue processing methods. (1) Tissue is dissociated and washed. (2) Tissue is cut into small pieces. (3a) In the enzymatic digestion method, the dissected tissue pieces are incubated in an enzyme-containing solution to digest the ECM and fully release EVs. The enzymatic digestion is terminated after a certain time, typically 15–30 min. (3b) In tissue culture method, the dissected tissue pieces are placed in culture medium and cultured under appropriate conditions, usually for 24–48 h. (4) The solution containing the tissue pieces is collected and passed through a cell strainer to remove large tissue segments. (5) Differential centrifugation is used to remove cells and cell debris, and the supernatant is collected and used to isolate EVs

Tissue processing methods generally include mechanical disruption, enzymatic digestion, and tissue culture. In the mechanical disruption method, a tissue homogenizer is usually used to homogenize the tissue, and then a series of centrifugation steps are performed on the tissue homogenate to remove cells and cell debris, and EVs are isolated following. Mechanical disruption is thought to result in cell broken and release of intracellular and other membrane vesicles, reducing the purity of Ti-EVs (Gupta et al. 2005, Mincheva‐Nilsson et al. 2016, Crescitelli et al. 2021). The processes of enzymatic digestion and tissue culture are shown in Fig. 1. Both methods have their own advantages and disadvantages. Enzyme digestion only takes a short time, typically 15–30 min. But this method is considered too aggressive and has the potential to affect the surface of cells and EVs, thereby altering the function of EVs (Garcia-Contreras et al. 2015). Tissue culture method is gentler and will not cause damage to cells and Ti-EVs. However, tissue culture takes a long time, generally 24–48 h. The prolonged tissue culture and changes in the in vitro environment of tissue cells may result in new EVs released from cells that are different from those in the in vivo environment. In contrast, enzymatic digestion obtains a snapshot of EVs in the tissue at the time of dissection (Crescitelli et al. 2021).

### Enzymatic digestion method

Enzymatic digestion can digest the ECM to release the embedded EVs for isolation. This method can be performed on fresh or frozen tissue to fully dissociate cells and EVs in the tissue, at the same time to ensure enzymes not damaging the proteins on the surface of cells and EVs.

The type and concentration of enzyme, and incubation conditions are the key parameters to influence the effectiveness of enzymatic digestion method, and the main parameters used in different studies are listed in Table 1. It can be found that there are many types of enzymes used for Ti-EVs isolation. Brain tissue generally uses type III collagenase (Vella et al. 2017) or papain (Perez-Gonzalez et al. 2012); tumor tissue mostly uses collagenase D (Lunavat et al. 2017); other enzymes include collagenase I (Chen et al. 2020) and IV (Ishiguro et al. 2019). Papain is often used to digest nervous tissue (Yousef et al. 2018). Gentle papain digestion does not trigger cell lysis, thereby avoiding potential contamination of Ti-EVs isolation by intracellular organelles and vesicles. Collagenases are capable of degrading native collagen by gently and selectively digesting the ECM with minimal damage to cells. Collagenase type III can be used for isolating cells from injury-sensitive tissues, like brain (Vella et al. 2017). Collagenase D is also used to dissociate cells from various tissues, like liver, adipocytes, islet (Uchea et al. 2015; Benck et al. 2016). Crescitelli combined collagenase D and DNase I to break down tumor tissue (Crescitelli et al. 2021). DNase I can reduce cell aggregation caused by extracellular DNA during cell dissociation (Möller et al. 2012; Legroux et al. 2015), therefore reducing the medium viscosity and leading to a higher EVs yield (Crescitelli et al. 2020). Ishiguro perfused the liver with an enzyme-containing medium to dissolve the extracellular matrix and dissociate intercellular junctions, and the cells and EVs were separated from the connective tissue after enzymatic digestion (Ishiguro et al. 2019). Collagenase concentrations ranging from 0.5 to 5 mg/mL were used, but the different collagenase concentration was found to have no significant effect on the production of EVs. And the optimal perfusion time is 7–8 min. Too long a perfusion procedure increases the risk of damaging the thin connective tissue within the liver, leading to liver injury or rupture of the portal vein; while shorter times lead to incomplete lysis and preserve hepatocyte contacts, resulting in incomplete EV release. This optimal protocol achieves the isolation of liver cells and tissue EVs with very little damage, reduces the risk of EVs being contaminated by impurities. However, its limitation is that some Ti-EVs may be washed away in the perfusate, resulting in loss of Ti-EVs. Enzymatic digestion methods should be adjusted when treating different type of tissues, for example, when a well-established method is to be used on bone tissue that has never been subjected to enzymatic digestion, some parameters should be optimized, such as the type and concentration of the enzyme, and the incubation time (Li et al. 2021). Huang found that the brains of mice and macaques are more sensitive to enzyme than human, so the digestion time should be shortened (Huang et al. 2020).

**Table 1:** Main parameters of the enzyme digestion method

Tissue	Enzyme type	Concentration of enzyme	Solvent of enzyme	Enzyme’s solution amount/tissue weight	Incubation conditions	Terminate digestion	References
− 80 °C frozen human brain	Collagenase type 3	50 U/ml	DPBS	8 μl/mg	25 °C, 20 min	Protease and phosphatase inhibitors and EDTA	Su et al. (2021)
− 80 °C frozen human brain Fresh mouse brain Fresh macaque brain	Collagenase type 3	75 U/ml	Hibernate-E	800 μl/100 mg	37 °C, 20 min for human; 15 min for mouse and macaque	PhosSTOP and complete protease inhibitor	Huang et al. (2020), Vella et al. (2017)
Frozen human brain Frozen mouse brain	Papain	20 U/ml	Earle’s balanced salt solution	3 ml/ 0.5 g human brain 3 ml/whole mouse brain	37 °C, 15 min	Hibernate-E with halt protease and phosphatase inhibitor cocktails	Muraoka et al. (2020a, b)
Frozen mouse brain	Papain	20 U/mL	Hibernate-A		37 °C, 20 min	Hibernate-A with protease and phosphatase inhibitors	Gomes et al. (2023)
Fresh or frozen murine hemi-brains	Papain	20 U/ml	Hibernate-E	3 ml/half brain	37 °C, 15 min	–	Perez-Gonzalez et al. (2012)
− 80 °C frozen mouse brain	Papain	1 mg/ml	Hibernate-E	10 ml/whole brain	37 °C, 20 min	–	Hurwitz et al. (2018)
Frozen macaque brain	Papain	20 U/ml	Hibernate-A	10 ml/500 mg	37 °C, 15 min	–	Yelamanchili et al. (2015)
Fresh or frozen mouse brains Fresh or frozen mouse lung tumor	Papain	1 mg/ml	Hibernate-E	10 ml/0.4–1.0 g	37 °C, 20 min	Protease and phosphatase inhibitors	Hurwitz et al. (2019)
Fresh mouse melanomas tissue	Collagenase DDNase	2 mg/mL 400 U/ml	–	–	37 °C, 30 min	Fresh medium	Lunavat et al. (2017)
Fresh melanoma metastatic tissue	Collagenase D DNase I	2 mg/ml 40 U/ml	RPMI-1640; DMEM showed no negative effects on the isolated EVs	2 ml/0.2 g	37 °C, 30 min		Crescitelli et al. (2021)
Fresh human melanoma lymph node or skin metastases	Collagenase D DNase I	2 mg/ml 40 U/ml	RPMI	–	37 °C, 30 min		Jang et al. (2019)
Fresh mouse MC38 colorectal tumors	Collagenase IV dispase DNase I	0.2 mg/ml 2 mg/ml 0.002 mg/ml	IMDM	–	37 °C, 20 min	Protease inhibitors	Cianciaruso et al. (2019)
Fresh human metastatic malignant melanoma	Collagenase D DNase I	2 mg/ml 40 U/ml	RPMI-1640	–	37 °C, 30 min	–	Crescitelli et al. (2020)
Fresh mouse liver, skeletal muscle, heart	Collagenase D DNase I	2 mg/ml 40 U/ml	RPMI-1640	1 ml/0.025 g	37 °C, 30 min	–	Matejovič et al. (2021)
Fresh human lung tissues	First step: dispase II working solution Second step: DNase I	3 U/ml 0.5 mg/ml	– RPMI-1640	1 ml/100 mg 10 ml	37 °C, 1 h RT, 20 min	–	Liu et al. (2022)
Fresh mouse lung tissue	Collagenase D DNase I	2 mg/mL 40 U/mL	RPMI-1640	–	37 °C, 30 min	–	Lässer et al. (2021)
Fresh mouse intestine	Collagenase type I	300 U/ml	HBSS	–	37 °C, 30 min	PBS with protease inhibitor cocktail	Chen et al. (2020)
Fresh mouse liver	Collagenase type IV	1 mg/ml	HBSS	–	Perfusion for 7–8 min for tissue digestion and dissociation	–	Ishiguro et al. (2019)

Not only the yield of EVs in the tissue was concerned, the viability of tissue cells and EVs is another aspect being investigated when applying different strategies of enzymatic digestion. Crescitelli compared three tissue processing methods to study the effect of enzyme on Ti-EVs isolation. Scheme 1 is without enzyme digestion; scheme 2 is to isolate EVs first, resuspend and then treat with collagenase D and DNase I, and then separate again by ultracentrifugation; scheme 3 is to directly treat tissue fragments with the same enzymes used in scheme 2, and then isolate EVs. The results showed that the best EVs yield was observed in scheme 3, indicating that the compactness of the tissue would hinder the release of EVs into the supernatant, resulting in low yield of EVs. The enzyme digestion method can effectively destroy the ECM, which is conducive to the release of EVs into the supernatant (Crescitelli et al. 2020). It has been reported that the molecules on cell surface are not affected by collagenase D (Autengruber et al. 2012). In addition, Crescitelli found the combination of collagenase D and DNase I had no effect on the viability of the cell line HMC-1, and had minimal effects on the surface molecules of cells and EVs. CD9 and CD63 expression on the surface of cells and EVs did not change significantly, while CD81 decreased slightly (Crescitelli et al. 2020). Liu used a two-step enzymatic digestion method to dissociate human and mouse lung tissue. First, dispase II working solution was dripped into the lungs of mice until the lung lobes were fully expanded, and then liquefied 1% agarose was added dropwise. After the agarose had fully gelled, the lungs were harvested and put into dispase II working solution and incubated for 1 h at room temperature. After digestion, the number of viable cells obtained from human and murine lung tissue were 96.2% and 94.2% of the total cells, respectively. This suggests that enzymatic digestion causes minimal damage to lung cells, which would greatly reduce contamination of Ti-EVs by intracellular vesicles (Liu et al. 2022).

### Tissue culture method

Fresh tissue must be used and generally cut into smaller pieces prior to tissue culture. The obtained fresh tissue should be put into the culture medium as soon as possible and cultured under appropriate conditions to promote the tissue cells to release EVs, which are then collected for EV isolation.

Tissue culture uses a protocol similar to cell culture. For example, using cell culture medium, with the addition of penicillin–streptomycin, and with or without fetal bovine serum (FBS). If FBS is used, exosome-depleted ones are generally recommended (Lunavat et al. 2017). In conventional practice, tissues are typically cultured within a carbon dioxide incubator at 37 °C. However, certain studies involve culturing tissues under conditions maintained at 4 °C (Jingushi et al. 2018; Tomiyama et al. 2021). Tissues need to be cut into small pieces to facilitate the delivery of nutrients and gases (O_2_/CO_2_) to all cells, and also to facilitate the release of EVs secreted by tissue cells to the medium. Dense tissues need to be cut into smaller pieces than loose mucosal tissues (Mincheva‐Nilsson et al. 2016). Never use mechanical and/or enzymatic disruption as cell disruption, for it may introduce unnaturally secreted EVs from multivesicular bodies (MVBs). Freshly obtained tissue is processed immediately, and shorter incubation times are recommended, although a shorter culture time may result in insufficient Ti-EV release as well as a lower EVs recovery rate. Otherwise, if the culture time is too long, the viability of tissue cells may decrease, and the composition of released EVs may be changed. An increase in dead cells may lead to a significant increase in EVs impurities. Jingushi and Tomiyama cultured tissue for only 1–2 h at 4 °C (Jingushi et al. 2018; Tomiyama et al. 2021). Mincheva cultured tissue at 37 °C for 24–48 h, and up to 72 h depending on the viability of the tissue cells (Mincheva‐Nilsson et al. 2016). Tong cultured human placenta tissue at 37 °C for up to 16–96 h (Tong and Chamley 2018). Lapeire demonstrated that 24-h in vitro culture had no effect on adipose tissue integrity, metabolic activity, and bioactivity (Lapeire et al. 2014). In addition to the culture time, some studies have also examined the atmosphere of tissue culture. Knowing that the human placenta developed under hypoxic conditions during the first 10 weeks of gestation, Tong investigated the effect of oxygen concentrations (2%, 8%, and 20%) in culture conditions on the release of EVs from the human placenta. The results showed that oxygen concentration did not influence the number and the mean and modal sizes of EVs, and the concentration of proteins in EVs (Tong et al. 2016).

Gomes compared both enzymatic digestion and tissue culture methods using mouse brain tissue. The tissue culture method provides a smaller total protein yield of EVs, but it shows a higher particle: protein ratio than the enzymatic digestion method, indicating higher EV purity. Proteomics results showed more proteins were identified in EVs from the tissue culture method (1660 proteins) compared to the enzymatic digestion method (409 proteins). In the category of cellular components “exosomes” (GO:0070062), EVs isolated from tissue culture could detect more proteins. While for the rest of the cell component category, the protein numbers looked similar for both methods (Gomes et al. 2023). The digestion with papain and/or other enzymes would affect the molecules on the plasma membrane surface (Parish et al. 1977; Iijima et al. 1999). This may be the reason that EVs proteins from tissue culture methods are particularly abundant in the membrane and extracellular domain categories. EVs isolated by tissue culture method are structurally, biochemically, and functionally intact (Gomes et al. 2023). However, Gomes used different isolation methods for enzymatic digestion and tissue culture when isolating Ti-EVs. Density gradient centrifugation was used for enzymatic digestion method, while a combination of size exclusion chromatography (SEC) and sucrose cushions was used for tissue culture. Isolation methods also have an impact on Ti-EVs; therefore, the differences among Ti-EVs cannot be attributed only to different tissue processing methods. Cianciaruso compared the two methods of enzyme digestion and non-enzyme digestion. From the nanoparticle tracking analysis (NTA) results, EVs number obtained by enzyme digestion was small and showed multiple peaks, while non-enzyme digestion showed a single peak. Therefore, it is considered that fewer but more heterogeneous EVs were obtained after enzymatic digestion. However, there was no significant difference in the western blot (WB) results of the two methods, both expressed EV characteristic proteins; however, both also expressed the endoplasmic reticulum protein GP96 (Cianciaruso et al. 2019). It should be noted that due to the differences in density among different tissues, attention must be paid when applying the above conclusions to other tissues. Most EVs isolated from adipose tissue have used tissue culture methods to process the tissue (Kranendonk et al. 2014a, b; Huang et al. 2021; Dong et al. 2022; Hong et al. 2022). When extracting EVs from the brain, enzymatic digestion methods are used more frequently, partly because brain samples are cryopreserved and are not suitable for tissue culture methods (Perez-Gonzalez et al. 2012; Muraoka et al. 2020a, b; Su et al. 2021).

### Other factors before tissue processing

Several factors during the process between tissue harvesting and tissue processing can also affect the isolation of Ti-EVs. After the tissue is obtained, perfusion using phosphate buffered saline (PBS) is beneficial to flush the blood cells and reduces the contamination of EVs. For blood-rich tissues, such as liver and heart, perfusion prior to tissue processing is critical. Liu perfused mouse lung tissue with cold PBS to remove blood before extracting Ti-EVs. Blood contamination in tissues influences cell viability, EVs recovery, and EVs proteome data (Liu et al. 2022). When isolating brain EVs, no significant difference in particle yield was observed after perfusion compared without perfusion, but the Golgi marker GM130 was significantly depleted, suggesting that perfusion played a role in reducing EVs contamination (Huang et al. 2020). Removal of blood to eliminate plasma EVs contamination through perfusion can be realized in animals, but not feasible when processing human samples.

EVs isolation typically takes a long time and may not be performed immediately after surgical harvesting of tissue. In addition, all the tissues needed for research are usually not available at the same time. For proteomics and genomics, it is recommended that all the tissue samples should be analyzed at the same time. Therefore, the harvested tissues need to be stored. Huang assessed the effect of the waiting time from death to treatment (post-mortem interval, PMI) on EVs isolation. After taking the macaque brain tissue, it was placed for 2, 6, or 24 h at room temperature before EVs collection. Compared with 2 h and 6 h, the EVs in the tissues resting for 24 h had a higher yield, but the number of intracellular proteins also increased significantly, indicating more cell destruction in the 24 h tissues. Long PMI did not affect the RNA biotype of EVs in macaque brain tissue, but resulted in the degradation of some small RNAs and lower miRNA diversity. For many, miRNAs are only detected in EVs from 2-h tissue (Huang et al. 2020). Perez-Gonzalez isolated EVs from human brain tissue frozen for several years, long-term frozen mouse brain, and freshly isolated mouse brain to evaluate the effects of cryopreservation on Ti-EVs, and transmission electron microscope (TEM) images showed no difference in morphology of isolated EVs (Perez-Gonzalez et al. 2012). However, this study did not present proteomics or RNA sequencing data; therefore, we cannot judge whether cryopreservation alters the purity and function of Ti-EVs or not. Some precious human tissues cannot be processed immediately after harvesting, but are cryopreserved. Although the tissue cell viability is preserved well in cold storage (Hyatt and Wilber 1959), the formation of ice crystals in storage temperature and the freeze–thaw cycles may destabilize EVs lipid membranes (Cheng et al. 2019). Using fresh tissue as a control, Shizhen Shen (Shen et al. 2023) compared the effects of − 80 °C frozen tissue and − 80 °C frozen tissue lysate on tissue EVs. After freezing at − 80 °C, the integrity of the cell membrane was damaged and intracellular vesicles were released. The production of both small EVs (sEVs) and large EVs (lEVs) was significantly increased, and markers associated with cell were slightly upregulated. In the − 80 °C frozen tissue lysate, lEVs numbers decreased slightly, while sEVs increased, and the expression of sEVs-related markers was also slightly downregulated. A portion of EVs proteins and miRNAs are lost in both approaches, but proteins are affected less than miRNAs. The authors prefer frozen tissues at − 80 °C to study the protein composition of Ti-EVs, as this storage method is simpler. Although tissue frozen can damage the cell membrane, this method did not significantly alter the EVs contents due to the intracellular vesicle origin of EVs. When studying miRNA components, it is recommended to use fresh tissue to isolate tissue EVs (Shen et al. 2023).

## Isolation of Ti-EVs

Ti-EVs isolation is more difficult than isolating EVs from cell supernatant and urine, as the medium used to isolate Ti-EVs usually contains more contamination like cell debris, intracellular vesicles, and intercellular components. Currently, there is no optimal method readily available for isolating Ti-EVs to meet experimental and clinical needs. It is challenging to design and implement an appropriate isolation method in which several factors need to be considered, including the expected yield, purity, integrity, and concentration of EVs, depending on downstream analysis and the scientific question to be addressed (Witwer et al. 2017).

The EVs isolation methods including ultracentrifugation (UC), density gradient centrifugation, SEC, immunoaffinity, PEG precipitation, asymmetric field flow fractionation, etc., are also described in other works (Coumans et al. 2017; Shao et al. 2018; Gandham et al. 2020). PEG precipitation method is a simple operation and of low cost, and does not require expensive equipment such as an ultracentrifuge (Karttunen et al. 2018). However, the EVs purity obtained by precipitation is quite low. Matejovič extracted EVs from liver tissue culture supernatants using a precipitation kit and UC. The Ti-EVs isolated by UC were observed as EV-like structures in TEM, while those isolated by precipitation mainly showed debris-like structures, but no EV-like. UC samples contained more EVs of smaller size than precipitation. The calnexin, a protein associated with endoplasmic reticulum, was detected in Ti-EVs isolated by precipitation, but not in UC samples, indicating that Ti-EVs isolated by precipitation contained non-EV contamination (Matejovič et al. 2021). UC is currently the most popular EVs isolation method (Gardiner et al. 2016). But using UC alone may lead to co-precipitation of protein aggregates. Adding a sucrose cushion purification step after UC can effectively reduce the contamination of these protein aggregates. Matejovič used a sucrose cushion to purify EVs after UC, which improved the yield, count, total protein content, and CD63 expression of Ti-EVs. EVs signature proteins HSP70, Alix, and TSG101 could be detected in Ti-EVs isolated both with and without sucrose cushion after UC, while CD63 was only detected in sucrose cushion purified samples. Furthermore, calnexin was detected in a higher level in EVs not purified by sucrose cushion (Matejovič et al. 2021). SEC can avoid aggregation of EVs during UC (Linares et al. 2015), and remove the soluble protein and lipoprotein that are often contaminated in EVs separated by UC or density gradient centrifugation (Karimi et al. 2018). Many studies have used SEC for the isolation of plasma EVs and achieved promising results (Gaspar et al. 2020; Guo et al. 2021). Muraoka performed UC to obtain EVs pellets from mouse brain tissue, and then conducted a EVs purification step either by a density gradient centrifugation or SEC. The results showed that higher EVs particle number and protein yield can be obtained by SEC. However, both NTA and TEM results demonstrated a broader size distribution of Ti-EVs isolated by SEC than that by density gradient centrifugation. Nano-LC–MS/MS analysis revealed that SEC-isolated Ti-EVs contained non-EVs proteins (Muraoka et al. 2020a, b). Therefore, the density gradient centrifugation is necessary for proteomic analysis of Ti-EVs (Muraoka et al. 2020a, b). Huang extracted EVs from human brain tissue and compared three isolation methods, i.e., density gradient centrifugation, SEC + UC, and SEC + ultrafiltration (UF). From the NTA results, Ti-EVs number obtained by SEC was slightly higher, which was consistent with the results of Muraoka’s study. However, the protein concentration of Ti-EVs was similar among the three different methods, thus resulting in a higher particle:protein ratio of Ti-EVs isolated by SEC than those by density gradient centrifugation. In the WB results, calnexin was only found in the SEC + UF lane, but not in the other two lanes, indicating that UC has a slight advantage over UF in improving the Ti-EVs purity after SEC. Although Ti-EVs isolated by the SEC + UC method had the highest particle:protein ratio, the authors do not recommend using SEC to replace density gradient centrifugation (Huang et al. 2020). Density gradient centrifugation is considered to obtain the highest purity of EVs (Onodi et al. 2018; Zhang et al. 2020a, b), and currently used most frequently in Ti-EVs isolation.

The key parameters in the density gradient centrifugation process include the density gradient medium, the separation of EVs by flotation or sedimentation, and the density layer collected, as listed in Table 2. Density gradient medium mainly included sucrose solution and iodixanol (Optiprep), and Kowal compared the purification effects of the two gradient mediums. When using iodixanol, the precipitate was mainly floating in two separate fractions with densities of 1.115 g/mL (fraction 3) and 1.145 g/mL (fraction 5). Both of these two fractions contain EV characteristic proteins MHC II, CD63 and CD9. TEM results revealed that the 1.115 g/mL fraction contained a majority of small EVs with a diameter of 50–150 nm. In contrast, sucrose density medium resulted in a series of continuous fractions containing MHC II and CD9 at densities from 1.12 to 1.19 g/mL, with most EVs floating in two adjacent fractions at 1.15 and 1.17 g/mL. Therefore, iodixanol could separate EVs subtypes of different densities and sizes, with more small EVs concentrated in the 1.115 g/mL light fraction. However, the sucrose gradient forms a continuous distribution of particles, which is not conducive to accurately distinguishing different EVs subtypes (Kowal et al. 2016). The difference in the separation of EVs between sucrose and iodixanol may be the result of different viscosity and osmolarity. The isoosmotic property of iodixanol is ideal for the isolation of cells and subcellular membrane vesicles (Li and Donowitz 2008). Hurwitz compared flotation and sedimentation methods to separate EVs. The flotation method is to resuspend the EVs pellet in a high-concentration iodixanol solution, and then place several low-concentration iodixanol gradients on top of it layer by layer; while the sedimentation method is to place the EVs sample on the top of the iodixanol gradient. TEM showed the presence of cellular debris in Ti-EVs isolated by sedimentation. While the floating Ti-EVs showed highly pure and abundant membrane-structured vesicles. Mass spectrometry identified 865 proteins in Ti-EVs isolated by the sedimentation method and 1,045 proteins in floating Ti-EVs. For the same amount of protein, the abundance of EVs proteins including CD81, Alix, Rab10, Flotillin, and CHMP4B was higher in the floating EVs, while the abundance of calnexin and ATP2A2 was relatively higher in the sedimentation samples. These findings suggest that Ti-EVs isolated by sedimentation are contaminated by membrane debris or/and other lipid particles that migrate together with Ti-EVs into the same density gradient (Hurwitz et al. 2018). The floating-based separation method can prevent protein aggregates from migrating up to the EVs density layer to improve the purity of EVs (Zonneveld et al. 2014; Kowal et al. 2016). We noticed that the density corresponding to the collected EV layers in different studies varied greatly, from 1.06 to 1.19 g/ml, as shown in Table 2. In addition to possible differences in individual laboratory testing methods for density, different tissue sources can also affect the density of Ti-EVs. Hurwitz compared Ti-EVs isolated from brain and tumor tissue, and found that tumor tissue had two distinct populations of Ti-EVs, 1.075 g/ml (low-density EVs) and 1.144 g/ml (high-density EVs), while brain Ti-EVs only enriched in low density (Hurwitz et al. 2019). EVs from different tissues may float to fractions with different densities, or may segregate into low- or high-density subpopulations. Crescitelli isolated large EVs by centrifugation at 16,500 g for 20 min, and small EVs by centrifugation at 118,000 g for 2.5 h from melanoma tissue. Both EVs showed that EVs characteristic proteins Flotillin-1, CD63, and CD81 were all positive, and calnexin is also positive at different degrees. The large and small EVs were further subjected to density gradient centrifugation, and the low-density fraction at 1.116 g/ml and the high-density fraction at 1.176 g/ml were collected, respectively. Proteomic analysis of these fractions revealed that particles enriched in primarily low-density small EVs were most similar to EXOs from the endosomal pathway. Proteins enriched in high-density small EVs are associated with the proteasome and nucleus. These proteins were precipitated during centrifugation at 118,000 g, but separated from lower density components during density gradient centrifugation (Crescitelli et al. 2020).

**Table 2:** Main steps in the isolation of Ti-EVs by density gradient centrifugation

Pre-centrifugate	Density gradient centrifugate	Re-pellet	Density of EVs fraction	Reference
4 °C, 300 g, 5 min; 4 °C, 2000 g, 10 min; 4 °C, 10,000 g, 30 min	Triple sucrose cushion (0.6 M, 1.3 M, 2.5 M); EVs were overlaid on the top; 4 °C, 200000 g, 173 min	4 °C, 128,000 g, 80 min	1.08 g/ml	Su et al. (2021)
4 °C, 300 g, 10 min; 4 °C, 2000 g, 10 min; 4 °C, 10,000 g, 10 min; 0.22um filter; 4 °C, 100,000 g, 70 min	Sucrose step gradient (2.0 M, 1.5 M, 1 M, 0.825 M, 0.65 M); EVs were resuspended in 0.475 M of sucrose solution and overlaid on the top; 4 °C, 200,000 g, 20 h	4 °C, 100,000 g, 70 min	1.10–1.15 g/ml	Muraoka et al. (2020a, b)
4 °C, 300 g, 10 min; 4 °C, 2000 g, 10 min; 4 °C, 10,000 g, 30 min; 4 °C, 100,000 g, 70 min, twice	6 sucrose solutions (0.25 M, 0.6 M, 0.95 M, 1.3 M, 1.65 M, 2.0 M); EVs were resuspended in 0.95 M sucrose solution 4 °C, 200,000°Cg, 16 h	4 °C, 100,000 g, 70 min	1.07–1.17 g/ml	Perez-Gonzalez et al. (2012), Yelamanchili et al. (2015)
4 °C, 500 g, 5 min; 4 °C, 2000 g, 10 min; 4 °C, 10,000 g, 30 min; 0.45 μm filter; 4 °C, 100,000 g, 2 h	Sedimentation gradient: (Hurwitz et al. 2016) Iodixanol solutions (40%, 20%, 10% and 5%); EVs were layered on the top 4 °C, 100,000 g, 18 h Floatation gradient: (Kowal et al. 2016) 60% iodixanol 1:1 mixed with EVs; 20% and 10% iodixanol were layered on the top; 4 °C, 268,000 g, 50 min	Sedimentation gradient: 4 °C, 100,000 g, 70 min Floatation gradient: 4 °C, 100,000 g, 2 h	1.07–1.09 g/mL	Hurwitz et al. (2018)
500 g, 5 min; 2000 g, 10 min; 4 °C, 10,000 g, 30 min; 4 °C, 110,000 g, 70 min; 4 °C, 134,000 g, 70 min	EVs were mixed with 90% sucrose stock solution to a final sucrose concentration 82% Overlay the sucrose gradient on top (70%, 64%, 58%, 52%, 46%, 40%, 34%, 28%, 22%, 16%, 10%);(Chiou and Ansel 2016) 4 °C, 100,000 g, 16 h	4 °C, 134,000 g, 70 min	1.14 g/ml	Cianciaruso et al. (2019)
4 °C, 500 g, 5 min; 4 °C, 2000 g, 10 min; 4 °C, 10,000 g, 40 min; 0.45 μm fiter; 4 °C, 100,000 g, 2 h	60% iodixanol 1:1 mixed with EV; 20% and 10% iodixanol were layered on the top; 4 °C, 268,000 g, 50 min	4 °C, 100,000 g, 2 h	Light EVs: 1.075 g/ml; Dense EVs: 1.144 g/ml	Hurwitz et al. (2019)
4 °C, 300 g for 10 min; 4 °C, 2000 g for 20 min; 4 °C, 16,500 g 20 min; 4 °C, 110,000 g for 2.5 h	Iodixanol gradient: EVs were mixed with 60% OptiPrep to a OptiPrep concentration of 45% Iodixanol gradient (35%, 30%, 28%, 26%, 24%, 22% × 2 and 20%); 4 °C, 178,000 g, 16 h Iodixanol cushion: EVs were mixed with 60% OptiPrep to a OptiPrep concentration of 45% 30% and 10% iodixanol solution were laid on the top; 4 °C, 178,000 g, 2 h	118,000 g, 3.5 h	Low density (LD): 1.111–1.121 g/ml; ~ 1.058–1.163 g/ml	Jang et al. (2019)
4 °C, 300 g, 10 min; 4 °C, 2000 g, 20 min; 4 °C, 16,500 g, 20 min (LEV); 4 °C, 118,000 g, 2.5 h (SEV)	Iodixanol gradient: EVs were mixed with 60% OptiPrep to a OptiPrep concentration of 45% Iodixanol gradient (35%, 30%, 28%, 24%, 22% × 2 and 20%); 4 °C, 186,000 g, 16 h Iodixanol cushion: EVs were mixed with 60% OptiPrep to a OptiPrep concentration of 45% 30% and 10% iodixanol solution were laid on the top; 4 °C, 186,000 g, 2.5 h		Low density (LD): 1.111–1.121 g/ml; High density (HD): 1.163–1.189 g/ml; ~ 1.058–1.163 g/ml	Crescitelli et al. (2021), Lässer et al. (2021)
4 °C, 500 g, 10 min; 4 °C, 1500 g, 15 min; 4 °C, 10,000 g, 30 min; 0.2um filter; 4 °C, 100,000 g, 2 h, twice	Iodixanol gradient (5%, 10%, 20% and 40%); EVs were overlaid on the top; 4 °C, 100,000 g, 18 h	4 °C, 100,000 g, 3 h	1.086–1.103 g/ml	Jeurissen et al. (2017)
1,000 g, 5 min; 10000 g, 30 min; 70,000 g, 60 min, twice	EVs were resuspended in 2.6 M sucrose; linear sucrose gradient (2.0–0.25 M sucrose, 20 mM Tris–HCl (pH 7.2)); 270,000 g, 16 h	70,000 g, 60 min	1.06–1.11 g/ml	Wang et al. (2008)
4 °C, 300 g for 20 min; 4 °C, 3000 g for 20 min; 4 °C, 10,000 g 60 min; 0.22um filter; 4 °C, 110,000 g, 70 min, twice	EVs were resuspended in 2.25 M sucrose solution and then poured on the top of 2.5 M sucrose; sucrose density gradient (2 M, 1.75 M, 1.5 M, 1.25 M, 1 M, 0.75 M, 0.5 M and 0.25 M); 4 °C, 180,000 g, 13 h	4 °C, 110,000 g, 70 min	1.05–1.14 g/ml	Liu et al. (2022)
4 °C, 2500 g, 30 min; 4 °C, 10,000 g, 35 min; 0.2-μm filter; 4 °C, 110,000 g, 2 h	Discontinuous iodixanol gradient (5%, 10%, 20%, and 40%). EVs were overlaid on the top 4 °C, 110,000 g, 2–5 h; or 4 °C, 100,000 × g, 16 h	4 °C, 110,000 g, 2 h	1.10–1.19 g/ml。	Mincheva‐Nilsson et al. (2016)
4 °C, 1000 g, 10 m in; 4 °C, 2000 g, 20 min; 4 °C, 5000 g, 30 min; 4 °C, 15,000 g, 1 h; 0.22um filter; 4 °C, 120,000 g, 130 min, twice	Optiprep iodixanol Solutions (40%, 20% and 5%); EVs were loaded on the top; 4 °C, 288,000 g, 5 h	4 °C, 100,000 g, 70 min	1.12–1.19 g/ml	Chen et al. (2020)
4 °C, 3000 g, 30 min; 4 °C, 10,000 g, 60 min; 4 °C, 100,000 g, 90 min, twice	Linear sucrose gradient (0.25–2.5 M in PBS); EVs were loaded on the top; 4 °C, 100,000 g, 18 h	–	1.13–1.19 g/ml	Lazar et al. (2015, 2016)
4 °C, 500 g, 10 min; 4 °C, 1500 g, 15 min; 4 °C, 10,000 g, 30 min; 4 °C, 100,000 g, 2 h, twice	Linear sucrose gradient (2.5–0.4 M sucrose, 20 mM Tris–HCL, pH 7.4); EVs was resuspended in 2.5 M sucrose; 4 °C, 190,000 g, 14–16 h	–	1.12–1.14 g/ml	Kranendonk et al. (2014a, b)

## Purity characterization of Ti-EVs

The complexity of Ti-EVs isolation makes the purity characterization of Ti-EVs necessary. The particle:protein ratio is a surrogate indicator of EVs purity (Webber and Clayton 2013). But this method is not reliable, since the number of particles measured by existing methods does not necessarily correspond to the number of EVs, and the co-separated impurity particles are also counted and taken as the number of EVs (Witwer et al. 2013; Bachurski et al. 2019). International Society of Extracellular Vesicles (ISEV) recommends protein characterization of isolated EVs (Witwer et al. 2017; Théry et al. 2018). The existence of EVs or EVs subtypes is proved by detecting EVs characteristic proteins; at the same time, the purity of EVs samples is evaluated by testing non-EVs and co-isolated impurity proteins. This section will mainly focus on the characterization of the characteristic proteins and impurity proteins of Ti-EVs.

### Western blot

WB is an effective method to characterize EV characteristic proteins and impurity proteins. Endosomal pathway EV protein markers, syntenin and TSG101, and tetraspanins, like CD81, CD63 and CD9, are usually used to prove the presence of EVs (Clotilde Théry et al. 2006, Mincheva‐Nilsson et al. 2016, Théry et al. 2018). However, the expression abundance of these markers may be different in EVs from different species and sources. Hoshino performed a large-scale, comprehensive analysis of the proteome of EVs from 426 human cancer and non-cancer samples from tissues, cells, and body fluids. Some commonly used EVs markers were expressed in low amounts in human plasma. This indicated the need to find new pan-EVs markers. Therefore, they screened 13 new pan-EVs markers from tens of thousands of human EVs proteins. Among them, A2M, B2M (Zagorac et al. 2012), STOM (Snyers et al. 1999; Mairhofer et al. 2002), FLNA, FN1, GSN, HBB, LGALS3BP, RAP1B, ACTB, and JCHAIN are proteins that are transported through endosomes (Hoshino et al. 2020). They all have high expression in all human EVs samples and can be used as new pan-EVs markers. ACTB, MSN (Muriel et al. 2016), and RAP1B (Pizon et al. 1994) represent markers for human EXOs and exomeres. STOM is found only in EXOs that can be used to distinguish EXOs from exomeres (Hoshino et al. 2020). Golgi’s protein BIP, endoplasmic reticulum protein calnexin, and mitochondrial protein VDAC, CYTOCHROME C, and ATP5A are often used as impurity proteins to judge the purity of EVs (Théry et al. 2018) (Bordas et al. 2020). When separating EVs from brain tissue, the impurity proteins include Histone H1 (from nucleus), HSPE1 (from mitochondria), RAB2A (from the Golgi apparatus) (Muraoka et al. 2020a, b), CytC, NSE (Gomes et al. 2023), Synaptophysin and SNAP-25 (Yelamanchili et al. 2015). In addition, Grp94 (Jayabalan et al. 2019), Lamp-1 (Deng et al. 2009), and GM130 (Zhou et al. 2020) are also used as negative control proteins when isolating EVs from adipose tissue. The platelet marker CD41a can be used to demonstrate the degree of blood contamination of EVsv (Crescitelli et al. 2020). When comparing the purity of Ti-EVs obtained by three different isolation methods, Huang found that the calnexin protein in the WB results was more credible than the particle: protein ratio of Ti-EVs in reflecting the purity of Ti-EVs (Huang et al. 2020). Vella separated EVs from human brain tissue by density gradient centrifugation, and obtained two EV-containing fractions, F2 and F3. The authors ran WB and used characteristic proteins to distinguish Ti-EVs in these two fractions, which belong to different EVs subtypes. Between them, F2 was highly enriched in endosomal protein syntenin and tetraspanin CD81, while there was no endosomal protein enrichment in F3, but the common EVs protein marker Flotillin-1 was detected. Therefore, it is speculated that the EVs in F3 are mainly of non-endosome origin, while those in F2 are mainly of endosome origin (Vella et al. 2017). Protein abundance may vary greatly between different species or different tissues; therefore, appropriate protein markers need to be carefully selected (Hoshino et al. 2020). Loyer extracted EVs from cardiac ischemic tissue and used cardiomyocyte troponin T as a characteristic marker of cardiac Ti-EVs (Loyer et al. 2018). GPRC5A and AGER were identified as signature proteins of EVs from lung tissue (Liu et al. 2022). Li detected high PLIN-A/B expression derived from the adipocyte, little CD68 from macrophage, and no CD31 from the endothelial cell in isolated adipose Ti-EVs, suggesting that these EVs are mainly produced by perivascular adipocytes (Li et al. 2019).

### Proteomic

In addition to WB, mass spectrometry-based proteomic analysis is often used to provide evidence of the purity of EVs. The first valuable analysis in proteomic is the protein number detected from EVs samples and the degree of overlap between the identified protein types and the EVs protein database (Ji et al. 2021), such as Vesiclepedia (Kalra et al. 2012), EVpedia (Kim et al. 2013), and Exocarta (Keerthikumar et al. 2016). Huang identified 427 proteins from human brain Ti-EVs. After comparing with Vesiclepedia, EVpedia, and Exocarta, 99% of the proteins coincided with the proteins in the database (Huang et al. 2020). Proteomics can also give more detailed information on EVs characteristic proteins and impurity proteins. Among the 1144 proteins identified by Kowal, there are proteins associated with endosomal pathway and trafficking, exosome biogenesis, and also the newly discovered small EVs markers ADAM10 and EHD1 (Kowal et al. 2016). EVs marker proteins identified by Huang include tetraspanins, cytoplasmic proteins, Annexins, RABS, and cytoskeletal proteins (Huang et al. 2020). Liu identified two characteristic proteins of lung Ti-EVs, GPRC5A and AGER, by proteomics. More cytoskeleton and cytoplasm proteins were identified from lung cells compared with Ti-EVs (Liu et al. 2022).

Gene Ontology (GO) can give bioinformatics information about the identified proteins, such as the source of cellular components of the proteins (Huang et al. 2009; Cheung et al. 2016). Cellular composition terms related to EVs include: “membrane-bound vesicle”, “extracellular vesicle”, “extracellular exosome”, “extracellular organelle”, and “extracellular region” (Crescitelli et al. 2020). Meanwhile, terms related to cellular debris or contaminants include: “nucleus”, “Golgi apparatus”, “endoplasmic reticulum”, “synaptic vesicles”, or “blood particles” (Vella et al. 2017) (Muraoka et al. 2020a, b). An abundance of proteins associated with terms such as “blood”, “platelets”, “red blood cells”, “plasma”, and “T cells” may indicate contamination from blood cell or infiltration of immune cells (Huang et al. 2020). Of note, proteins associated with mitochondria are generally considered contaminants. However, Jang found that three mitochondria inner membrane proteins were highly expressed in Ti-EVs isolated from melanoma metastatic tissue rather than non-melanoma tissue, and considered them to be potential biomarkers (Jang et al. 2019). Huang compared the proteomics of brain homogenate (BH), 10 K precipitated large vesicles (10 K), and purified EVs. The GO terms related to EVs like “exosomes”, “cytoplasmic vesicle”, and “vesicle” were enriched in both 10 K and purified EVs, and common terms like “cytoskeleton”, “lysosome”, and “cytoplasm” were detected in all three groups. “Plasma membrane”, “membrane”, and “whole membrane” were only detected in purified EVs, while “intracellular part”, “protein containing complex”, “nucleolus”, and “nucleosome” were found in both 10 K and BH (Huang et al. 2020). Crescitelli isolated lEVs from melanoma tissue by centrifugation at 16,500 g for 20 min, and sEVs by centrifugation at 118,000 g for 2.5 h. The lEVs and sEVs were further purified by density gradient centrifugation, and the low-density fraction of 1.116 g/ml and the high-density fraction of 1.176 g/ml were collected, respectively. Proteomic analysis was performed on six groups of lEVs, sEVs, low-density lEVs, high-density lEVs, low-density sEVs and high-density sEVs. The 742 proteins that were differentially expressed by multiple group comparisons were divided into 6 clusters. Proteins from clusters 1 and 2 were enriched in low-density sEVs and low-density lEVs, and had the highest proportion of membrane proteins, 77% and 86%, respectively. GO terms for these two clusters are associated with “extracellular exosomes”, “plasma membrane”, “endosomes”/“circulating endosomes”, and “Golgi apparatus”. Clusters 3, 4, and 5 were enriched in lEVs, and contained proteins from “endoplasmic reticulum” or “mitochondria”. Cluster 6 contained proteins enriched in sEVs and high-density sEVs, and was associated with the “cytosol”, “proteasome”, and “nucleoplasm”. DnaJ homology subfamily C member 13 (also known as RME-8), a protein associated with membrane trafficking through early endosomes (Chang et al. 2004; Girard et al. 2005; Fujibayashi et al. 2008), was only enriched in low-density sEVs. These results suggest that mainly low-density sEVs are most similar to EXOs from the endosomal pathway. High-density sEVs-enriched proteins associated with the proteasome and nucleus were co-pelleted during centrifugation at 118,000 g, but separated from lower density fractions by density gradient centrifugation (Crescitelli et al. 2020) (Table 3).

**Table 3:** Purity characterization of Ti-EVs

Tissue	WB		Proteomic	Reference
	EVs marker proteins	Non-EVs proteins		
Human brain	Syntenin, TSG101, CD81, Flotillin-1	Bip and calnexin are undetected; VDAC is low level	EVs marker proteins: CD63, CHMP4B, CHMP6, VPS26, VPS29, VPS50, PDC6IP, STX12, STX7, SORT1, ADAM10 and EHD1; GO terms associated with EVs: “membrane-bounded vesicle”, “extracellular region”, “extracellular organelle”, “extracellular vesicle” and “extracellular exosome”	Vella et al. (2017)
Human brain Mouse brain	CD9, CD63, CD81, TSG101, syntenin	Calnexin and GM130 are undetected in human samples; GM130, calnexin and Bip can be found in mouse samples	EVs marker proteins: CD81, CD9, FLOT1, FLOT2, ANXA11, ANXA3, ANXA4, RAB14, RAB1A, ACTN1; GO terms associated with EVs: “exosomes”, “vesicle” and “cytoplasmic vesicle”; GO terms associated with contaminations: “platelet”, “blood”, “plasma”, “T-cell”, “fibroblast”, and “erythrocyte”	Huang et al. (2020)
Human brain	–	–	GO terms associated with contaminations: “nucleus”, “mitochondria”, “ER”, and “Golgi-related proteins”	Muraoka et al. (2020a, b)
Human brain Mouse brain	CD9, CD81, Annexins, LAMP2	Histone H1, HSPE1, calnexin, RAB2A	EVs marker proteins: CD9, CD81, CD82, PDCD6IP; Alix, HGS Non-EVs proteins: IMMT, calnexin, and Bip	Muraoka et al. (2020a, b)
Mouse brain	CD81, Flotillin-1, EEA1	Cytochrome C (CytC) and neuron-specific enolase (NSE)	EVs marker proteins: CD9, CD81; GO terms: approx. 40% relative enrichment of “the extracellular region categories” and approx. 15% relative enrichment of “synaptic part”	Gomes et al. (2023)
Mouse brain	Alix, HSC70, CD63, TSG101, CD81, Rab8a	Calnexin	EVs marker proteins: CD81, Alix, Rab10, Flotillin proteins, and charged multivesicular body protein 4B (CHMP4B) Non-EV proteins: calnexin and ATP2A2;	Hurwitz et al. (2018)
Mouse brain Mouse lung tumor	Alix, HSC70, CD63, TSG101, Flotillin-2, syntenin-1, CD81	Calnexin	GO terms associated with EVs: “exosomal”, “lysosomal”, and “plasma membrane proteins”	Hurwitz et al. (2019)
Melanoma lymph node or skin metastases	Flotillin-1	Calnexin	EVs marker proteins: CD9, CD81, CD63, Syntenin-1, and Flotillins; Three mitochondrial inner membrane proteins (COX6c, SLC25A22, and MT-CO2) were highly expressed in Ti-EVs from melanoma metastatic tissue	Jang et al. (2019)
Fresh human metastatic malignant melanoma	flotillin-1, CD63, CD81, CD9; ADAM10 (present in small EVs); Mitofilin (present in large EVs)	Calnexin; CD41a, platelet marker	EVs marker proteins: ADAM10 and EHD4 GO terms associated with EVs: “Extracellular exosome”, “Plasma membrane”, “Endosome”/ “Recycling endosome” and “Golgi apparatus”; GO terms associated with contaminations: “Endoplasmic reticulum” or “Mitochondrion”	Crescitelli et al. (2020)
Human melanoma, colon cancer and colon mucosa; mouse melanoma and colon cancer	CD63, CD9, CD81, Flotillin-1; ADAM10; Mitofilin	Calnexin	EVs marker proteins: TSG101, RAB proteins, annexins and Flotillin-1, cannot be used to distinguish small and large EVs Small EVs marker: ADAM10 and EHD4; Large EVs marker: Mitofilin	Crescitelli et al. (2021)
Fresh human tumor tissue; peritumoral non-involved tissue	Galectin-3-BP, Moesin, Stomatin, syntenin-1, β2-microglobulin, CD9, TSG101, CD81		Several standard exosome markers, including CD63, TSG101, Flotillins, and ALIX, were not enriched in human plasma Pan-exosome/exomere markers: RAP1B. ACTB, moesin (MSN) and RAP1B; Specific exosome marker: STOM	Hoshino et al. (2020)
Fresh human and murine lung tissues	GPRC5A, AGER (lung specific EV markers); ALIX, TSG101, CD9, CD81, Flotillin-1;	GM130, calnexin, TOM20, VDAC1	GO terms associated with EVs: “extracellular exosome,” “plasma membrane” and “cytosol”	Liu et al. (2022)
Fresh mouse MC38 colorectal tumors	Alix, CD63, TSG101, CD81, CD9;	Calnexin GP96	Non-EVs proteins: RNA-associated proteins;	Cianciaruso et al. (2019)
Fresh mouse lung tissue	Flotillin-1		GO terms associated with EVs: “Extracellular exosome” and “Membrane” with 33% and 58% of the identified proteins	Lässer et al. (2021)
Human placentae	Cytokeratin and PLGF (trophoblasts);	Vimentin (stromal cells); CD45 (leucocytes); glycophorin A (red blood cells)	Non-EVs proteins: CD31 and CD47	Tong et al. (2016)
Mouse mesenteric adipose tissue	PLIN-A and PLIN-B (adipocyte marker); ALIX, CD63, TSG101 and CD9;	CD68 (macrophage), CD3 (endothelial cell marker)	–	Li et al. (2019)
Human visceral and subcutaneous adipose tissue	CD81, CD9, CD63	GRP94	GO associated with EVs: “exosomes”, “lysosome”, “cytoplasm”, “plasma membrane”, and “extracellular (region, space, and matrix)”; EVs marker proteins: CD63, CD9, CD81, syntenin-1, TSG101; small EVs markers: ADAM10 and EHD4	Tamara et al. (2022)
Rat inguinal fat pads	CD9, CD63, TSG101, ALIX	–	GO analysis: the majority of sEV-AT proteins resided in the cytoplasm (31%) and membrane (21%). Only 7% of proteins were annotated as secreted proteins	Zhang et al. (2020a, b)

## Research progress of Ti-EVs in tumors

Currently, Ti-EVs used in tumor diagnosis and treatment research are mainly derived from tumor and adipose tissue. Therefore, in this paper, we only discuss the research progress of tumor- and adipose-derived Ti-EVs in tumors.

### Ti-EVs derived from tumor tissue

The tumor microenvironment plays an important role in the occurrence and development of tumors, and EVs are an important part of the tumor microenvironment. EVs have been proven to be involved in multiple processes of tumorigenesis and development. At present, numerous studies have revealed the relationship between tumor Ti-EVs and the occurrence and development of various cancers (Li et al. 2021; Qin et al. 2021), including the promotion of tumor cell proliferation, angiogenesis, tumor migration and metastasis, and immune microenvironment regulation by Ti-EVs.

Jingushi found that LAIR1 was highly expressed in renal cell carcinoma (RCC) Ti-EVs compared with that in adjacent non-cancerous renal tissue. LAIR1 activated the Akt pathway by upregulating the phosphorylation status of Akt, thereby increasing the cell proliferation of RCC. And using siRNA to knock down LAIR1 can reduce RCC cell proliferation by inhibiting Akt phosphorylation, providing a new target for clinical treatment of RCC (Jingushi et al. 2019). Zhang found that gastric cancer Ti-EVs induced neutrophil autophagy and tumor precursor activation through the HMGB1/TLR4/NF-κB signaling pathway, promoting cancer cell proliferation and migration. This result provided new insights into how neutrophils regulated and promoted tumor growth and metastasis, and also provided new strategies for the diagnosis, treatment, and prognosis of tumors (Zhang et al. 2018). Bone-metastatic RCC Ti-EVs contained higher APN and thus a higher pro-angiogenic capacity compared with Ti-EVs from non-bone metastatic tumor (Takeda et al. 2023). Colorectal cancer-derived Ti-EVs were enriched in CAT1 and may promote angiogenesis through activation of the arginine-NO-cGMP metabolic pathway and ERK/p38 phosphorylation signaling in vascular endothelial cells. This may also provide new drug pathways for colorectal cancer treatment (Ikeda et al. 2021). Ti-EVs from clear cell renal cell carcinoma (ccRCC) regulated the permeability of the vascular endothelial cell layer and promoted endothelial migration of ccRCC cells in an AZU1-dependent manner (Jingushi et al. 2018). Eldh indicated that 69 of the most abundant proteins in bladder Ti-EVs were enriched in cancer-related metabolic pathways, and were associated with poor prognosis (Eldh et al. 2021). Specific damage-associated molecular pattern (DAMP) molecules of tumor Ti-EVs may induce immunosuppression and pro-tumor inflammation (Hoshino et al. 2020). Cianciaruso found that TAM-EVs promoted T cell proliferation and interferon gamma (IFNγ) production in vitro. Several proteins in TAM-EVs were involved in the biosynthesis of arachidonic acid (AA) lipid metabolites. TAM-EVs may shift the AA catabolism to a COX1-dependent pathway instead of a COX2-dependent pathway, which may limit the tumor-promoting effects of certain prostaglandins (PGs) (Cianciaruso et al. 2019). Lunavat found miR-211-5p was significantly upregulated in cells and EVs isolated from tumor tissues after vemurafenib treatment. Mechanistically, inhibition of BRAF by vemurafenib modulated PERK1/2 and MITF pathways, resulting in upregulation of TRPM1, which induced the expression of miR-211-5p. Upon activation of this pathway, the survival pathway of melanoma cells was also activated, thereby promoting resistance to vemurafenib. Inhibition of miR-211-5p in vemurafenib-resistant cell lines negatively affected cell proliferation, which provides a solution for vemurafenib resistance (Lunavat et al. 2017).

Compared with body fluid- and cell line-derived EVs, Ti-EVs derived from specific tissues provide advantages in good specificity and easy analysis of the spatiotemporal heterogeneity of the tissue microenvironment. Moreover, Ti-EVs mediate complex intercellular communication between tissue cells, and therefore carry more original information. Meanwhile, by having a pair of EVs with the same genetic background from tumor and nearby normal tissues, comparative analysis can be conducted to help accurately identifying cancer-specific cargo in tumor Ti-EVs without considering the noise of individual differences. Tumor Ti-EVs have great potential to be used as biomarkers for cancer detection in early stage, as well as for diagnosing unknown primary tumors (Li et al. 2021). Hoshino performed a large-scale analysis of the extracellular vesicles and particles (EVP) proteome, comparing with EVP isolated from adjacent and distant tissues, protein markers were screened from EVP of tumor tissue, with a sensitivity and specificity of more than 90%. Some of these protein markers are also present in plasma EVP. By detecting tumor-associated plasma EVP in various cancer patients from stage I to stage IV, it was shown that plasma EVP protein can be used as biomarkers to detect cancer in early stage. In addition, EVP protein packaging differed in different types of tumor and reflected tumor biology. For example, proteins associated with epithelial–mesenchymal transition (EMT), coagulation, and actin signaling pathways were enriched in pancreatic adenocarcinoma EVP, while cell cycle, metabolism, and RNA processing pathways were highly expressed in lung adenocarcinoma EVP. Specific combinations of EVP proteins from plasma or tumor tissue were able to distinguish cancers of unknown origin. Therefore, EVP profiles from tissue biopsies can help classify cancer types, thereby support more personalized treatment plans for cancer patients with unknown origin of the primary tumor (Hoshino et al. 2020).

Melanoma is a highly aggressive malignancy with high rates of metastasis and mortality, and its prevalence keeps increasing. Ti-EVs isolated from melanoma, with a higher expression of mitochondrial membrane proteins, were different from cell-line-derived EVs. Jang isolated EVs from melanoma tissue and discovered two proteins from mitochondrial membrane, MT-CO2 and COX6c, which were also found in the plasma of melanoma, breast, and ovarian cancer patients, therefore can be potential biomarkers for liquid biopsy (Jang et al. 2019). Crescitelli isolated EV subpopulations from metastatic melanoma tissues, studied 34 melanoma-related genes, and found that 6 genes (BRAF, STK19, CDKN2A, PPP6C, NRAS, and RAC) were mutated in Ti-EVs. Ti-EV DNA showed a higher frequency of mutant alleles when compared with the total plasma DNA, indicating the potential value of Ti-EVs as melanoma biomarkers (Crescitelli et al. 2022). Colorectal cancer ranks third among common cancers and second among cancer-related deaths. Ikeda found that CAT1 expression was higher on tumor Ti-EVs than on normal Ti-EVs. In addition, the concentration of EV-CAT1 in the plasma of colorectal cancer patients was also significantly higher compared with healthy donors. Importantly, EV-CAT1 levels have been significantly increased even in phase I patients, indicating that EV-CAT1 levels had high potential in colorectal cancer detection. The combined diagnostic model of EV-CAT1 and carcinoembryonic antigen had ideal detection efficiency (sensitivity 66.7%, specificity 92.0%, AUC value 0.907; 95%CI 0.850–0.963) (Ikeda et al. 2021). Ji selected five cases of colorectal cancer that recurred within 1 year as the recurrence group, and five cases that did not relapse within 1 year as the non-recurrence group. In the adjacent tissue of the recurrence group, four Ti-EVs proteins (HLA-DPA1, S100P, NUP205, PCNA) were significantly expressed, so these proteins can be used as biomarkers to predict postoperative recurrence (Ji et al. 2021). Bladder cancer (BCa) is the cancer most closely associated with urine. Tomiyama performed simultaneous proteomic analysis on both tumor Ti-EVs and urinary EVs, and found that most proteins detected in tumor Ti-EVs were also found in urinary EVs. Three EVs proteins, HSP90, SDC1, and MARCKS, were identified as reliable biomarkers for BCa detection (Tomiyama et al. 2021). Eldh found 69 proteins in EVs released from bladder tissue that were associated with poor prognosis after transurethral bladder resection (TUR-B), 20 of which were also found in urinary EVs. These 20 proteins had a greater impact on long-term survival and were able to be considered as potential prognostic markers (Eldh et al. 2021). ccRCC is the most common histopathological type of sporadic cancer, accounting for approximately 90–95% of renal cancer cases. Jingushi discovered that AZU1 in ccRCC-derived EVs had great potential as a biomarker and can be detected through non-invasive liquid biopsy (Jingushi et al. 2018). Compared with normal tissues and their Ti-EVs, CA9, CD70, and CD147 were increased in tumor tissues and their Ti-EVs. So, CA9, CD70, and CD147 may be used as biomarkers for identifying RCC (Himbert et al. 2020). Non-small cell lung cancer (NSCLC) is the main type of lung cancers. Song isolated Ti-EVs from cisplatin-resistant and cisplatin-sensitive tumor tissues and compared the expression levels of miR-4443 between them. Compared with cisplatin-sensitive Ti-EVs, miR-4443 levels were upregulated in cisplatin-resistant Ti-EVs, and thus could serve as a predictive marker of tumor resistance (Song et al. 2021). Ovarian clear cell carcinoma (OCC) is a cancer caused by endometriomas. Maruoka isolated Ti-EVs from OCC and normal ovarian tissue from the same patients, and preoperative and postoperative serum samples were also collected from OCC and atypical endometrial hyperplasia (AEH) patients (control group). A total of 959 miRNAs were studied, and 6 OCC tissue-specific miRNAs were identified, which can be used as biomarkers for their early diagnosis (Maruoka et al. 2022).

### Ti-EVs derived from adipose tissue

Obesity may increase overall cancer risk and be associated with worse outcomes in cancer patients (Booth et al. 2015). The adipose tissue of obese individuals secretes large amounts of EVs, which mediate information delivery between adipocytes and cancer cells (Lazar et al. 2016). Adipose Ti-EVs have been proven to play a key role in the link between obesity and cancer.

Adipose Ti-EVs can promote tumor cell proliferation and cell death resistance. Breast cancer cells (ZR75.1) treated with Ti-EVs derived from cancer-associated adipose had a higher degree of phosphorylation of cAMP response element-binding protein (CREB) serine residue 133, indicating that Ti-EVs can promote the breast cancer cell proliferation (Jeurissen et al. 2017). Similarly, extracellular signal-regulated kinases (ERK) phosphorylation was increased after treatment with obese adipose Ti-EVs, which promoted the proliferation of MCF-7 cells (Ramos-Andrade et al. 2020). EVs derived from adipose-derived stem cells (ADSCs) facilitate tumor proliferation and growth by regulating Wnt/β-catenin signaling, through the procollagen galactosyltransferase 2 (COLGALT2) pathway (Wang et al. 2020). Wei Ying isolated macrophages from adipose tissue. Adipose tissue macrophages (ATMs) in lean mice were mainly anti-inflammatory, while the number of ATMs in obese mice increased and showed a pro-inflammatory activation state. The levels of miR-155 increased in ATM-EVs from obese mice, promoting obesity-induced insulin resistance (Ying et al. 2017). miR-155 has also been shown to play oncogenic/anti-apoptotic effects in breast cancer cells (Zhang et al. 2013). Mathiesen found that PC3ML metastatic prostate cancer cells proliferated significantly after contacting with EVs from adipose tissue. This may be attributed to the transcription factor twist family bHLH transcription factor 1 (twist1), which is critical for secondary tumor growth (Mathiesen et al. 2023).

Adipose Ti-EVs can promote tumor cell migration and invasion. Obese adipose Ti-EVs induced the migration and invasion of MDA-MB-231 cells by promoting the increase of protein kinase B (PKB, namely AKT) phosphorylation (Ramos-Andrade et al. 2020). Lazar observed that adipose Ti-EVs isolated from obese mice promoted migration of melanoma cell more than those isolated from lean mice. The results were also observed in human adipose tissue taken from people with different body mass index (BMIs). Adipose Ti-EVs derived from overweight and obese people increased melanoma migration compared with those from the lean individuals (Lazar et al. 2015). miRNAs contained in adipose Ti-EVs, such as miR-128 and miR-155, were also found to promote cancer invasion and metastasis, especially in a pro-inflammatory environment (Mathiesen et al. 2023). Moraes found that ADSC-derived EVs may activate the Janus kinase (JAK)/signal transducer and activator of transcription (STAT-3) pathway through epidermal growth factor receptor 1 (EGFR-1)/IL-6, thereby promoting breast cancer cell migration and metastasis (Moraes et al. 2021). Khanh confirmed that ADSCs-EVs from type 2 diabetes mellitus (T2DM) patients induced higher expression of genes associated with breast cancer cell (BCC) migration and metastasis. Thus, treatment with these EVs significantly increased BCC migration in vitro and induced higher lung metastasis in vivo (Khanh et al. 2020).

Adipose Ti-EVs can promote angiogenesis. EVs derived from ADSCs contain pro-angiogenic proteins. Internalization of ADSC-EVs by endothelial cells significantly increased their migration and proliferation in vitro and induced angiogenesis in mice (Gangadaran et al. 2021). Wang found that EVs derived from ADSCs expressed more miR-132 after treatment with vascular endothelial growth factor-C (VEGF-C). The transfer of miR-132 by ADSCs-EVs to lymphatic endothelial cells (LECs) promoted the proliferation and migration of LECs and tube formation (Wang et al. 2018). Moraes also suggested that ADSCs secrete EVs enriched in miR-132 and miR-31 to induce angiogenesis in obesity-associated cancers (Moraes et al. 2021). Li found that miR-221-3p was highly enriched in obese perivascular adipose tissue (PVAT) and its derived EVs. miR-221-3p can significantly promote the proliferation and migration of vascular smooth muscle cells (VSMC) (Li et al. 2019).

Adipose Ti-EVs can also communicate with immune cells and change the immune microenvironment to promote tumor progression. ADSC-EVs deliver STAT3 to macrophages, increase Arg-1 expression in macrophage, and promote M2 subtype polarization to reduce white adipose tissue (WAT) inflammation, which significantly reduces pro-inflammatory TNF-α secretion but increases anti-inflammatory IL-10 secretion (Zhao et al. 2017). In tumors, M2 polarization of macrophages and upregulation of anti-inflammatory cytokines will create an immunosuppressive tumor microenvironment and promote tumor growth. Kranendonk found that adipocytes and macrophages achieve cross-talk through EVs. Macrophage-driven AT inflammation reduces insulin signaling in adipocytes, leading to systemic insulin resistance (IR) and increased cancer risk (Kranendonk et al. 2014a, b). Deng verified that adipose Ti-EVs significantly enhanced the development of IR, impaired glucose tolerance, and induced inflammatory cytokines in a toll-like receptor 4 (TLR4)-dependent manner, which would increase cancer incidence (Deng et al. 2009). Blazquez found that ADSC-EVs significantly inhibited the proliferation and differentiation of CD4 and CD8 T cells, and also inhibited the secretion of IFN-γ. At the same time, there was a lack of major histocompatibility complex (MHC-II) class molecules and costimulatory molecules, which were beneficial to the survival and development of tumors (Rebeca Blazquez et al. 2014).

Adipose Ti-EVs can regulate the tumor cell metabolism. Through the transfer of the functional enzymes ECHA and hydroxyacyl-CoA dehydrogenase (HCDH), adipose Ti-EVs may induce metabolic reprogramming in favor of fatty acid oxidation (FAO) in recipient cells. The metabolic reprogramming was associated with increased mitochondrial number and density in melanoma and prostate tumor cells (Lazar et al. 2016). In addition, adipocyte EVs transported fatty acids (FA) to melanoma cells (Clement et al. 2020). These FAs were stored in lipid droplets and provided fuel for FAO. High-fat diet (HFD)-EVs delivered more FA, which increased lipid accumulation. And fueling FAO by FA may reshape the mitochondrial network in melanoma cells, redistributing these organelles to cell ends, thereby promoting cell migration. Liu found that adipose Ti-EV caused metabolic reprogramming of estrogen receptor + breast cancer cells, enhanced the dependence of breast cancer cells on mitochondrial respiration, and drove the proliferation of breast cancer cells (Liu et al. 2023).

The study of adipose Ti-EVs in the mechanism of tumor development will help discover new targets that inhibit tumor growth and provide new strategies for tumor treatment. For example, pharmacological inhibition of FAO completely reversed the effects of adipose Ti-EVs on obese tumor cell migration. Therefore, FAO inhibitors can be used for antitumor treatments, especially for obese patients (Lazar et al. 2016). ADSC-EVs can significantly reduce the apoptosis rate of neutrophils and significantly improve the phagocytic ability of neutrophils (Mahmoudi et al. 2019). These data consistently demonstrate that ADSC-EVs can bias the neutrophils to N1-like profile shift, which may provide a new strategy for cancer treatment. Zhou observed that there was more miR-424-5p in ADSC-EVs than the EVs from other MSCs. miR-424-5p can mediate PD-L1 repression by binding to 30-untranslated regions (UTR) in MM231 cells. MM231 cells exposed to ADSC-EVs were co-cultured with activated PBMCs, and the content of pro-inflammatory signals IFN-γ, IL-6, and TNF-α in the medium was increased, while the anti-inflammatory signal IL-10 decreased. Therefore, the antitumor effects of miR-424-5p-enriched ADSC-EVs were observed both in vitro and in vivo (Zhou et al. 2021). Treatment with miR-424-5p-enriched ADSC-EVs can induce cytotoxic T cells in tumors, which is a useful tumor treatment strategy. Li found that two subtypes of ADSCs (high CD90 and low CD90) had different antitumor activities. Compared with CD90-high ADSC-EVs, the latter significantly slowed down proliferation and inhibited migration of tumor cells (Li et al. 2020). Antitumor effects were exhibited in a breast cancer mouse model, suggesting that ADSC-EVs could be used as new effective therapeutic agent or drug delivery vesicles.

## Prospects

Isolation of highly pure Ti-EVs is a prerequisite for subsequent research, and the most desirable isolation of EVs from tissue is the efficient release of Ti-EVs from ECM confinement without causing any damages to tissue cells and EVs. However, the isolation process may deteriorate because of many factors involved, such as different sources of contamination. Any severe pressure (chopping, vortexing, homogenization) during tissue isolation and processing may cause cell rupture in the tissue, resulting in membrane debris of broken cells and the release of immature intracellular vesicles. If enzymatic digestion is used, over-digestion will destroy proteins on the surface of tissue cells and EVs. Therefore, it is necessary to optimize the parameters during enzymatic digestion, such as enzyme type, concentration, and digestion time. Freeze–thaw cycle and ice crystal formation may also damage EV membrane structure during the storage of the tissue and Ti-EVs at low temperature. Therefore, fresh tissue is recommended to use to isolate Ti-EVs, and if freezing is necessary, try to avoid multiple freeze–thaw cycles. Neighboring tissue cells not removed and residual blood cells can lead to EVs contamination from non-specific tissue cells. Therefore, removal of adjacent tissue and washing of tissue are effective methods to reduce these contaminants.

Although the research around Ti-EVs has received extensive attention in recent years, related investigation techniques and outcomes have also made great progress as reviewed above (Qin et al. 2021). However, there are still some issues to be elucidated in the isolation and characterization of Ti-EVs. For example, whether cryopreservation after specific tissue acquisition has a side effect on the isolation and application of Ti-EVs and how the tissue can be preserved to minimize this effect; whether enzyme treatment and tissue incubation cause a large difference in the applied research of Ti-EVs and which method may better reflect the real function of Ti-EVs. Finally, specific tissue processing and Ti-EVs isolation methods need to be investigated for different tissues as well as the most suitable EVs signature protein and potential impurity protein are necessary to be determined for characterizing the isolated EVs in question.

The role of Ti-EVs in the occurrence and development of tumors has been increasingly revealed. These studies have provided new ideas and strategies for early diagnosis, prognosis, and treatment of tumors. Due to the advantages of Ti-EVs over cell line and body fluid-derived EVs in tumor diagnostics, Ti-EVs have become a hot research topic in this field. Unlike the significant progress achieved by Ti-EVs in tumor diagnostics and prognosis research, there remains a lack of research on their role in tumor treatment. Several obstacles have hindered the progress of tissue EVs in oncological treatment research. For example, most human tissue is difficult to obtain, and EVs secreted by animal tissues may trigger immune response due to carrying exogenous molecules. Although tumor tissues and obese adipose tissues are relatively easier to access, the EVs they secrete pose a risk of promoting tumor growth. ADSCs are easily isolated from adipose tissue discarded during liposuction or other surgeries, and these ADSCs can be cultured in vitro and generate large numbers of ADSC-EVs. And there are already many studies proving that ADSC-EVs are effective potential therapeutic vehicles for tumors. However, EVs derived from ADSCs have been reported to possess a dual nature, with both tumor-promoting and antitumor effects, which is associated with the heterogeneous subtypes of ADSCs themselves. Therefore, screening ADSC subtype with antitumor properties from healthy donors’ adipose tissue as a source for therapeutic EVs is recommended. Alternatively, employing suitable stimuli on ADSCs to produce antitumor EVs could also be a feasible approach. Furthermore, some studies have demonstrated that loading anticancer drugs can further enhance the antitumor effects of ADSC-EVs. Anyway, ADSC-EVs have the therapeutic potential for tumor treatment.

## Data Availability

Not applicable.

## Abbreviations

EVs: Extracellular vesicles
EXOs: Exosomes
MVs: Microvesicles
ABs: Apoptosis bodies
ILVs: Intraluminal vesicles
MVEs: Multivesicular endosomes
Ti-EVs: Tissue-derived EVs
AD: Alzheimer's disease
ECM: Extracellular matrix
FBS: Fetal bovine serum
MVBs: Multivesicular bodies
SEC: Size exclusion chromatography
NTA: Nanoparticle tracking analysis
WB: Western blot
PBS: Phosphate buffered saline
PMI: Post-mortem interval
TEM: Transmission electron microscope
sEVs: Small EVs
lEVs: Large EVs
UC: Ultracentrifugation
UF: Ultrafiltration
GO: Gene ontology
RCC: Renal cell carcinoma
ccRCC: Clear cell renal cell carcinoma
DAMP: Damage-associated molecular pattern
IFNγ: Interferon gamma
AA: Arachidonic acid
EVP: Extracellular vesicles and particles
EMT: Epithelial–mesenchymal transition
BCa: Bladder cancer
NSCLC: Non-small cell lung cancer
OCC: Ovarian clear cell carcinoma
AEH: Atypical endometrial hyperplasia
ERK: Extracellular signal-regulated kinases
ADSCs: Adipose-derived stem cells
ATMs: Adipose tissue macrophages
BMIs: Body mass index
BCC: Breast cancer cell
LECs: Lymphatic endothelial cells
PVAT: Perivascular adipose tissue
VSMC: Vascular smooth muscle cells
WAT: White adipose tissue
IR: Insulin resistance
FAO: Fatty acid oxidation
FA: Fatty acids
HFD: High-fat diet
